# Training in Emergency Obstetrics: A Needs Assessment of U.S. Emergency Medicine Program Directors

**DOI:** 10.5811/westjem.2017.10.35273

**Published:** 2017-12-05

**Authors:** Daniel W. Robinson, Michael Anana, Mary A. Edens, Marc Kanter, Sorabh Khandelwal, Kaushal Shah, Todd Peterson

**Affiliations:** * University of Chicago, Department of Medicine, Section of Emergency Medicine, Chicago, Illinois; † Rutgers New Jersey Medical School, Department of Emergency Medicine, Newark, New Jersey; ‡ Louisiana State University Health Sciences Center – Shreveport, Department of Emergency Medicine, Shreveport, Louisiana; § Lincoln Medical and Mental Health Center, Department of Emergency Medicine, Bronx, New York; ¶ The Ohio State University, Department of Emergency Medicine, Columbus, Ohio; || Icahn School of Medicine at Mount Sinai, Department of Emergency Medicine, New York, New York; # University of Alabama at Birmingham, Department of Emergency Medicine, Birmingham, Alabama

## Abstract

**Introduction:**

Obstetrical emergencies are a high-risk yet infrequent occurrence in the emergency department. While U.S. emergency medicine (EM) residency graduates are required to perform 10 low-risk normal spontaneous vaginal deliveries, little is known about how residencies prepare residents to manage obstetrical emergencies. We sought to profile the current obstetrical training curricula through a survey of U.S. training programs.

**Methods:**

We sent a web-based survey covering the four most common obstetrical emergencies (pre-eclampsia/eclampsia, postpartum hemorrhage (PPH), shoulder dystocia, and breech presentation) through email invitations to all program directors (PD) of U.S. EM residency programs. The survey focused on curricular details as well as the comfort level of the PDs in the preparation of their graduating residents to treat obstetrical emergencies and normal vaginal deliveries.

**Results:**

Our survey had a 55% return rate (n=105/191). Of the residencies responding, 75% were in the academic setting, 20.2% community, 65% urban, and 29.8% suburban, and the obstetrical curricula were 2–4 weeks long occurring in post-graduate year one. The most common teaching method was didactics (84.1–98.1%), followed by oral cases for pre-eclampsia (48%) and PPH (37.2%), and homemade simulation for shoulder dystocia (37.5%) and breech delivery (33.3%). The PDs’ comfort about residency graduate skills was highest for normal spontaneous vaginal delivery, pre-eclampsia, and PPH. PDs were not as comfortable about their graduates’ skill in handling shoulder dystocia or breech delivery.

**Conclusion:**

Our survey found that PDs are less comfortable in their graduates’ ability to perform non-routine emergency obstetrical procedures.

## INTRODUCTION

Nationally, pregnancy-related conditions are the sixth most common reason for admission to the emergency department (ED) and the fourth most common final diagnosis from the ED for women of childbearing age.^1^ Pregnant women often present to the ED because they feel they are having an emergency.^2^ Being able to manage both emergent and non-emergent pregnancies is a hallmark of an emergency physician (EP).^2^ Currently there are no formal standardized teaching requirements beyond the minimum mandatory requirement of 10 low-risk, normal spontaneous vaginal deliveries^3^ (NSVD) and the assumption that obstetrical knowledge is a core principle of emergency medicine (EM), which means that EPs must achieve this knowledge in order to practice after residency.^3^,^4^

Obstetrical emergencies in emergency medicine (EM) are high risk for both the practitioner as well as the patient since it is one of the leading causes of maternal mortality.^5^ There is little evidence on malpractice claims related to deliveries performed in the ED; however, both obstetrics and EM are recognized as higher- risk specialties.^6^,^7^ Likewise, there is scant data about the rate and types of obstetrical emergencies that a recent EM graduate is likely to face. The few published studies are old and may not be representative of the current landscape.^8^,^9^ Recently it has been shown that current EM residents feel unprepared for management of these emergencies once they leave residency.^10^

The current Accreditation Council for Graduate Medical Education (ACGME) Review Committee for EM requires residents to demonstrate competency in key procedures, which includes vaginal deliveries. Residents are required to complete 10 low-risk NSVDs.^3^ In addition, the 2016 *Model of the Clinical Practice of Emergency Medicine* includes both normal pregnancy as well as complications of pregnancy, and labor and delivery and the postpartum period as a part of the core content of EM.^4^ As there have been no studies to date evaluating the incidence of either NSVD or complications of labor and delivery in EDs,^2^ it is not clear whether current requirements adequately prepare residents for independent practice in these areas.

Little is known about the current methods being used to teach EM residents about obstetrical and gynecologic emergencies. Anecdotal reports suggest that most use a combination of didactics and simulation. Simulation-based medical education (SBME) has been shown to be beneficial in many aspects of medical education.^11^–^15^ Likewise, the use of simulation has proven beneficial in teaching obstetrics/gynecology residents and family medicine residents the necessary skills to manage obstetrical emergencies.^5^ We sought to profile the present obstetric training curricula in U.S. EM residency programs through a survey of residency program directors (PD).

## METHODS

We developed a survey instrument (Table 1) based on a review of the literature on obstetrical emergencies.^2^,^5^,^8^–^10^ To keep the survey brief, we limited our inquiry to the four most common obstetrical emergencies based on author opinion: pre-eclampsia/eclampsia, postpartum hemorrhage (PPH), shoulder dystocia, and breech presentation. We felt that these four emergencies were the most commonly encountered obstetric pathologies in the ED and the most relevant to practicing EPs.

While participants were queried about program demographics, they were not asked any questions that would identify their program. We queried PDs about the allocation of curriculum time for obstetrical training, and the teaching methods used with the four most common obstetrical emergencies named above. Ultimately, the PDs were asked to rate their level of comfort with their graduating residents’ competence in managing these four obstetrical emergencies as well as their competence in performing a NSVD. Response options were four-point Likert-type scales where 1 = “very uncomfortable,” 2 = “uncomfortable,” 3 = “comfortable,” and 4 = “very comfortable.”

The survey was piloted by multiple associate program directors (APD) at each of the authors’ home institutions prior to distribution. Results of the pilot suggested minor changes for clarity and readability, which were incorporated into the final survey. We chose APDs to pilot so as not to bias the responses by having subjects answer multiple similar surveys. PD contact information was captured through the ACGME and FRIEDA Online® databases.^16^,^17^ Using REDCap,^18^ an electronic data collection tool, we distributed the survey anonymously to the PDs of all U.S. EM residency programs accredited by the ACGME. In November 2016, we sent an invitation email with a link to the online survey to the 191 PDs. We sent follow-up reminders once a week for three weeks. The study received institutional review board (IRB) approval by the University of Alabama at Birmingham. We compiled and analyzed data with Microsoft^®^ Excel.^19^

## RESULTS

We received 105 responses from 191 PDs who were sent the survey (55% return rate). Table 2 provides the characteristics of the responders. We found that of the directors surveyed, most were in academic and urban settings with a 2–4 week rotation in the PGY1 year. The most common teaching modalities (Figure) used for all types of obstetrical complications were didactics (84.1–98.1%). Oral cases were the second most common teaching method for pre-eclampsia (48%) and PPH (37.2%), while homemade simulation cases were the second most common teaching method for shoulder dystocia (37.5%) and breech delivery (33.3%). The PDs’ level of comfort (Figure) with their residency graduates was highest for NSVD, followed by pre-eclampsia and PPH. They were least comfortable with their graduates’ management of shoulder dystocia or breech delivery.

## DISCUSSION

To satisfy the requirement for 10 NSVDs, most of the EM programs we surveyed require residents to spend as few as two but as many as four weeks on an OB rotation during the PGY-1 year. PDs were comfortable with their residency graduates’ competence in managing NSVDs, pre-eclampsia/eclampsia, and PPH. However, they were not comfortable with their graduates’ competence in managing shoulder dystocia or breech deliveries. Anecdotal accounts report that these procedures are rare in the clinical environment.

Prior research has focused on residents,^10^ whereas our research focused on the perceived comfort levels of PDs. PDs were our focus as we felt that they would have the best understanding of their program’s curriculum. Future opportunities include surveying recent residency graduates to assess their actual comfort level with obstetric emergencies in clinical practice.

The predominant method for teaching labor and delivery complications are didactic sessions, with a small percentage using oral board cases and homemade simulation models. The findings of this survey indicate that although there is some variability in educational methods, most programs are using the same instructional methods for teaching obstetrical emergencies. Yet PDs are not comfortable with their graduates’ competence in managing two of the most complicated emergencies: shoulder dystocia and breech deliveries. Additional research is needed to better understand EM residency graduates’ experiences in treating obstetrical emergencies during their independent practice as well as their perceived competence in those areas. These results suggest that more rigorous teaching methods are needed to prepare residents for these uncommon yet serious obstetrical emergencies. They may also suggest the need for more rigorous program training requirements.

When assessing the PDs’ level of comfort with their graduating residents’ ability to treat obstetrical emergencies, our survey addressed only five conditions that we judged were the most important. Further research must be performed to establish whether our determination was accurate, or if other obstetrical emergencies, such as third trimester bleeding, perimortem cesarean section, and proficiency in performing an episiotomy, should be evaluated.

The nature of some of the obstetric emergencies that we queried, namely pre-eclampsia, eclampsia, and PPH, overlap significantly with other general medical conditions (GMC) that EPs treat (seizures, blood pressure management, hemorrhagic shock, etc.). PDs were comfortable with their graduating residents’ competencies that overlapped with commonly seen GMCs, whereas they were not comfortable with their residents’ competence in managing conditions that do not overlap with GMCs (e.g., shoulder dystocia and breech delivery). Likewise, the latter are more procedural in nature and more difficult to address with didactic teaching methods.

Breech deliveries are a rare procedure to perform for obstetrical residents and the likelihood that an EM resident would have an opportunity to participate in one during residency would be extremely rare.^20^ Therefore, their only education would be based on didactics and simulation. The authors recognized the rarity of such events, but felt that the stakes are as high for EPs as they are for obstetrical physicians. The fact that a breech delivery is rare does not protect EPs from needing to know how to care for the patient. Future research should focus on surveying residency graduates to establish the most common obstetrical conditions seen after graduation.

## LIMITATIONS

The main limitation of this study was our inability to query our population with more rigorous survey methods. Due to the concern about our collection of sensitive program information, specifically a rating of an EM resident graduate’s competence in managing complicated obstetrical cases, our IRB required that our data be collected anonymously. This limited our ability to track survey respondent participation and verify that our respondents were actually PDs. However, assuming that we connected with the correct population, we believe that the provision of anonymity provided assurances to our respondents that they could answer our questions honestly without concern for their responses being revealed.

A second limitation is the study’s response rate of 55%, which equates to a margin of error of 6.4%. To achieve an appreciably lower margin of error, the response rate would need to be considerably higher.

When assessing the PD’s comfort level in their graduating residents’ ability to treating obstetrical emergencies, our survey addressed only five of the most common and important obstetrical conditions. To keep the survey manageable in length, we did not include other less-common obstetrical emergencies such as third trimester bleeding, peri-mortem cesarean section, or proficiency in performing an episiotomy. Future research will need to survey residency graduates to establish the most common obstetrical conditions seen after graduation.

A final limitation was that we did not ask PDs about the types of obstetrical services available at their hospital training sites. This could potentially impact how much residents were exposed to obstetrical emergencies in the ED, as well as the PD’s confidence in their residents’ skills.

## CONCLUSION

Our findings show that PDs do not feel comfortable in their graduates’ competence in performing non-routine emergency obstetrical procedures. Follow-up research is planned to evaluate EM graduates’ experience with obstetrical emergencies in practice after residency training.

## Figures and Tables

**Figure:**
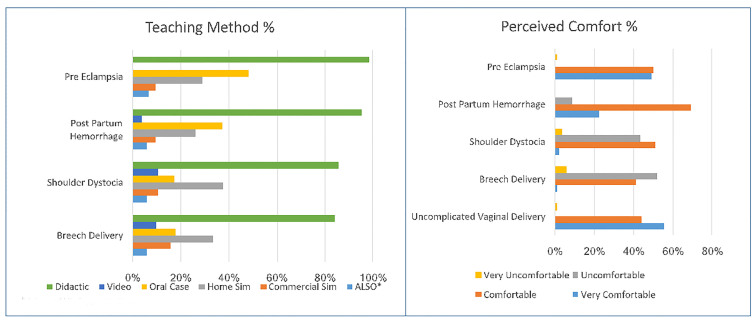
Teaching methods of obstetrical complications and program director’s perceived comfort of graduates abilities.

**Table 1:** Survey instrument based on the most common obstetrical emergencies.

•	What type of EM training program do you direct? PGY1-3/PGY1-4	
•	Which of the following best describes the residential setting of your residency program?	
	rural/urban/suburban	
•	Which of the following best describes the healthcare setting of your residency program?	
	academic/community/other	
•	Briefly describe the healthcare setting (open question)	
•	How many weeks are residents required to train during their OB/GYN rotation?	
	PGY1 0/1/2/3/4/>5	
	PGY2 0/1/2/3/4/>5	
	PGY3 0/1/2/3/4/>5	
	PGY4 0/1/2/3/4/>5	
•	Select the methods used to train your residents to treat the OB complications and difficult deliveries listed below (select all that apply).	
	Pre-eclampsia/eclampsia	Didactic/video/oral case/home sim/commercial sim/ALSO^*^
	Post-partum hemorrhage	Didactic/video/oral case/home sim/commercial sim/ALSO^*^
	Shoulder dystocia	Didactic/video/oral case/home sim/commercial sim/ALSO^*^
	Breech presentation	Didactic/video/oral case/home sim/commercial sim/ALSO^*^
•	How comfortable are you in your graduating residents’ ability to take care of the following OB emergencies?	
	Pre-eclampsia/eclampsia	Very uncomfortable/uncomfortable/comfortable/very comfortable
	Post-partum hemorrhage	Very uncomfortable/uncomfortable/comfortable/very comfortable
	Shoulder dystocia	Very uncomfortable/uncomfortable/comfortable/very comfortable
	Breech presentation	Very uncomfortable/uncomfortable/comfortable/very comfortable
	Normal vaginal delivery	Very uncomfortable/uncomfortable/comfortable/very comfortable

*ALSO*, Advanced Life Support of Obstetrics; *OB*, obstetrics, *GYN* gynaecology, *PGY,* post-graduate year; *Sim,* simulation.

**Table 2:** Characteristics of EM program directors who responded to survey regarding obstetrics/gynecology curriculum.

Characteristics of survey responders	% (n)
Residency program duration	
3 Years	71.4 (75)
4 Years	28.6 (30)
Program location	
Urban	65.4 (68)
Suburban	29.8 (31)
Rural	4.8 (5)
Healthcare setting	
Academic	75.0 (78)
Community	20.2 (21)
Other	4.8 (5)
Timing of OB/GYN rotation in curriculum	
PGY 1	84.9 (90)
PGY 2	12.3 (13)
PGY 3	2.8 (3)
PGY 4	0 (0)
Duration of OB/GYN rotation	
1 week	0.9 (1)
2 weeks	38.0 (41)
3 weeks	18.5 (20)
4 weeks	42.6 (46)

*OB*, obstetrics, *GYN* gynaecology, *PGY,* post-graduate year.
